# Double or hybrid diabetes: A systematic review on disease prevalence, characteristics and risk factors

**DOI:** 10.1038/s41387-019-0101-1

**Published:** 2019-11-04

**Authors:** Jomana Khawandanah

**Affiliations:** 1 Clinical Nutrition Department, Faculty of Applied Medical Sciences, King Abdulaziz University, Jeddah, Saudi Arabia; 2 Section for Nutrition Research, Department of Metabolism, Digestion and Reproduction, Faculty of Medicine, Imperial College London, London, United Kingdom

**Keywords:** Diabetes, Obesity

## Abstract

Diabetes mellitus is a worldwide epidemic affecting the health of millions of people. While type 1 diabetes (T1D) is caused by autoimmune destruction of the insulin-producing beta cells of the pancreas, type 2 diabetes (T2D) results from a combination of insulin resistance and beta cell insulin secretory defect. Clear definition and diagnosis of these two types of diabetes has been increasing more and more difficult, leading to the inclusion of a new category, namely double or hybrid diabetes (DD) that demonstrates symptoms of both T1D and T2D via the accelerator hypothesis. In this review, we discuss the worldwide prevalence of DD, its main physiological characteristics, including beta-cell autoimmunity, insulin resistance, and cardiovascular disease, the main risk factors of developing DD, mainly genetics, obesity and lifestyle choices, as well as potential treatments, such as insulin titration, metformin and behavioural modifications. Increasing awareness of DD among the general population and primary care practitioners is necessary for successfully treating this complex, hybrid disease in the future.

## Introduction

Diabetes mellitus is a chronic metabolic disease that is defined by persistent increased blood glucose levels (fasting blood glucose ≥ 126 mg/dl, random plasma glucose ≥ 200mg/dl, HbA1c ≥ 6.5%)^1^ leading at higher risk to serious and chronic microvascular and metabolic complications of type 1 diabetes (T1D) and the macrovascular complications of type 2 diabetes (T2D)^2–7^. The world prevalence of diabetes in adults was 6.4% (285 million people) in 2010, and are expected to raise to 7.7% by 2030^8^. Of course, ethnicity-dependent differences are expected^9,10^.

T1D (insulin-dependent) results in the destruction of the insulin-producing beta cells of the pancreas^7^. The cause of T1D is not clearly defined yet, but there is evidence for not only strong genetic predisposition, but also for environmental triggering, leading to complete dependence on daily insulin injections or pump and specialised medical care^11^. T1D results in the presence of autoantibodies against glutamic acid decarboxylase (GAD/GAD65), islet cells, insulin (IAA), protein tyrosine phosphatase-related islet antigen 2 (IA2/IA2β) as well as zinc transporter protein (ZnT8A) in the blood of these patients^12^. T1D is one of the most common metabolic/endocrine diseases diagnosed in children (80–90% of diabetic children)^13^; as an example, more than 3 million patients suffered from T1D in US in 2010, corresponding to 1 in 300 by 18 years old^14^. Global epidemiological studies have demonstrated that the incidence of T1D has been increasing to 2–5% annually^12^. On the other hand, the most common type in adults is T2D (non-insulin-dependent) that appears when the body develops resistance to insulin^7^, however there is also an recently increasing presence of young-onset T2D in children and adolescents^15^. T2D is a major metabolic disorder, which is characterised by increased blood sugar as a result of insulin resistance and due to reduced insulin secretion from pancreatic beta cells. Unhealthy dietary habits, obesity, genetic factors and a sedentary lifestyle are known to be the key risk factors for T2D development. Globally, around 5.1 million people between the ages of 20 to 79 died of T2D in the year 2015, which accounts for nearly 9% of overall mortality for this age group^16^. The increased consumption of dietary energy in comparison with low energy expenditure, resulting in obesity and weight gain is the major risk factor.

According to the first World Health Organization (WHO) global report on diabetes an outstanding number of 422 million adults live with this Non-Communicable Diseases (NCD) worldwide^17^. This number has almost made fourfold since 1980, which is largely because of the rise in T2D and other associated conditions including obesity, causing 1.5 million deaths due to heart attack, stroke, kidney failure or blindness in 2012 alone^17^. Specifically, the Kingdom of Saudi Arabia (KSA) was at the top 3 countries for diabetes prevalence in 2010, with an increased 18.9% prevalence projected for 2030^8^. Similarly, in children and adolescents of various ethnic groups worldwide, the prevalence of diabetes can range between 0.2–1.2%, with T1D being the most common type^18^. This increase is observed in the general Middle-East population and is mainly due to the nutrition transition associated to fast economic development, lifestyle changes reduced physical activity and escalated obesity^19,20^. In the Saudi National Diabetes Registry, all-cause mortality rate was ~17 per 1000 person-years, greater in men and older individuals, and associated with longer duration of diabetes, macrovascular complications, retinopathy, neuropathy, hypertension etc.^21^.

## T1D or T2D?

**Fig. 1:**
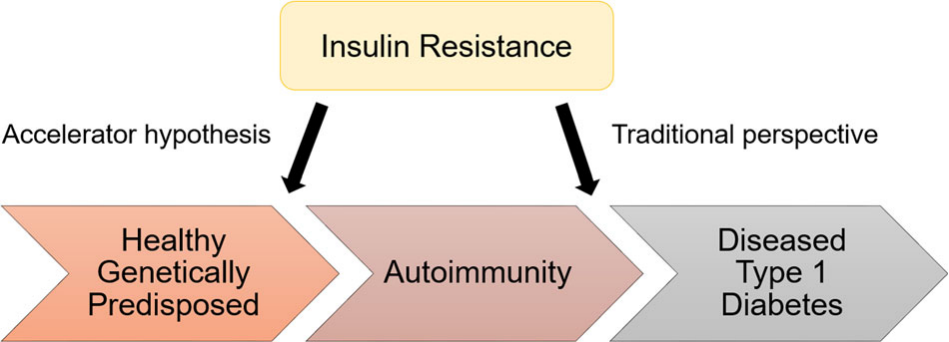
Qualitatively comparing the most prominent clinical features of T1D and T2D

Initial clinical observations in the ‘70s resulted in separating diabetes mellitus in two distinct forms—T1D defined by a defective immune system (*autoimmunity)*, and T2D defined by loss of insulin responsiveness (*metabolic syndrome)*^22^. Losing the control of blood glucose can result in beta cells being unable to secrete insulin, in tissues resisting to its action, or both. Classifying a clinical condition is very important in disease diagnosis and treatment as it can guide clinicians to translate scientific understanding to clinical practice^23^. Each classification can be further sub-divided depending on severity, and can be differently treated, ranging from insulin injections to lifestyle interventions^23^. However, distinguishing between T1D and T2D has increasingly become more difficult in terms of clinical characteristics and aetiology, as they both share beta-cell inefficiency. There is evidence that supports the hypothesis that T1D (*fast diabetes*), similarly to T2D (*slow diabetes*), will eventually cause insulin resistance, with body mass playing an important role^24^. Therefore, rather than expressing diabetic patients’ clinical variation into distinct categories, one could consider the diabetic spectrum/continuum as a dimensional factor^23^ (Fig. 1). Clinicians have already raised doubts regarding the classification of young patients with classical features of T1D (antibodies and ketoacidosis), but with obesity and a history of T2D in the family^25^. In an early case report of a 5-year-old boy, the co-existence of both types of diabetes was evident, with a single classification not possible; a condition that was taken into account during diagnosis and treatment^26^. Other examples include a case of a 13-year-old obese girl with elevated levels of blood glucose and beta-cell antibodies that was treated with insulin injections instead of blood glucose-reducing drugs, and another 13-year-old girl with T1D, but with excess weight resulting in insulin insensitivity, treated with metformin additionally to her very high insulin dosages^27^.

### The ‘accelerator hypothesis’

**Fig. 2:**
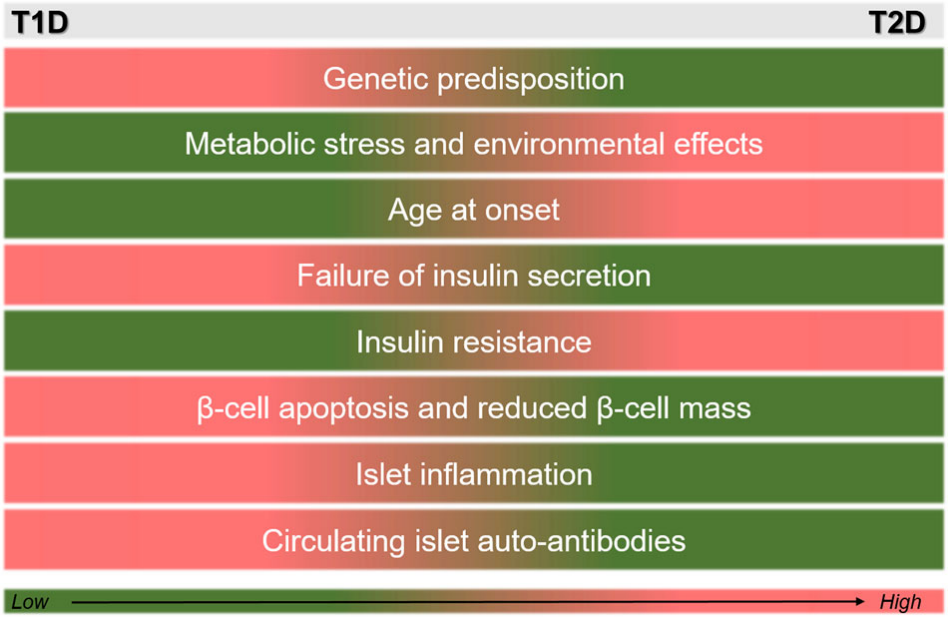
The role of insulin resistance in the development of diabetes phenotype (adapted from Stene, 2016)

The ‘accelerator hypothesis’ was first described in 2001 and argues that T1D and T2D are one and the same disorder, but distinguishable by the measure and tempo of three accelerators, one being intrinsic and two being acquired^28^. The accelerators include firstly beta cell death, important for diabetes development, secondly insulin resistance, caused by weight gain, visceral fat and sedentary lifestyle, and thirdly, beta cell autoimmunity (immune damage), driven by genetic factors^29^ (Fig. 2). Testing the accelerator hypothesis in children in the United Kingdom showed that the age of diagnosis of T1D is correlated with adiposity and higher body mass index (BMI), with strong gender-specific effects, as boys presented with T1D at a significantly earlier age^30^. Epidemiological data in many European countries proposed that children with T1D are increasingly becoming heavier, and with higher waist circumference, at age of disease onset, suggesting the importance of environmental accelerators in T1D development^31^. However, this observation was not confirmed in children of other ethnic groups (South Asian and Australian), suggesting that body fat composition, rather than BMI, might be a better measure of insulin resistance^32^. While several studies implicate the role of obesity during childhood as a risk factor for developing T1D, the association remains weak with undetermined causality^33^; therefore, more studies are necessary prior to testing this hypothesis in practice for T1D prevention^34^.

### The phenomenon of double diabetes

**Fig. 3:**
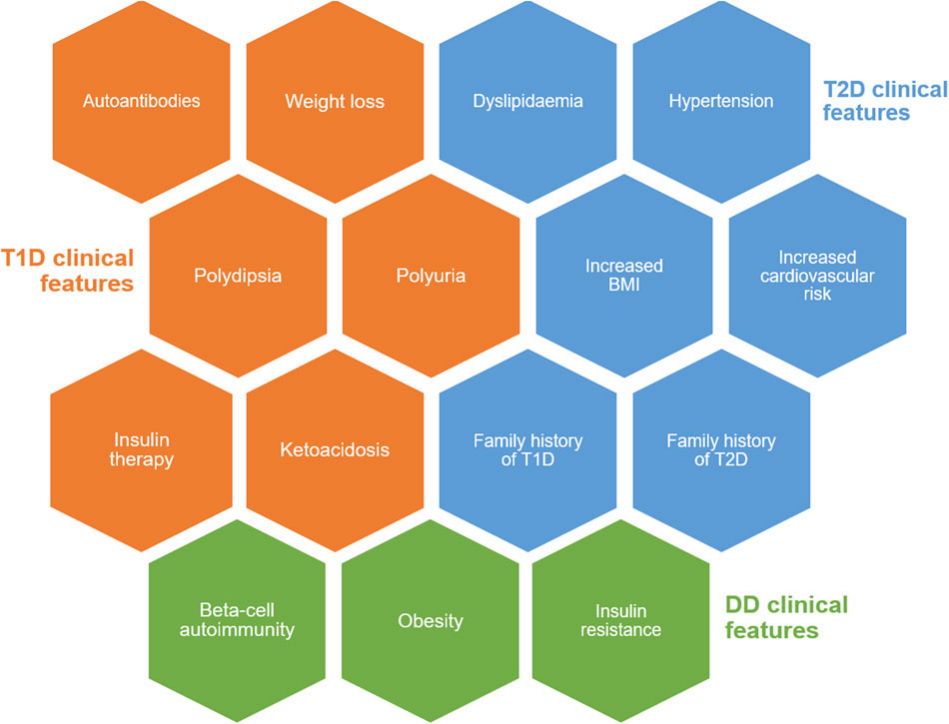
How to diagnose DD

The term ’double diabetes’ (DD) refers to the cases where the patient demonstrates characteristics as a result of a mixture of T1D and T2D (Fig. 3)^35^. Merger et al. found that in a large epidemiological study showed that a total of 25.5% of patients suffering from T1D additionally presented the metabolic syndrome^36^. Similarly, in a study including youth onset diabetes patients from east Delhi and the neighbouring Indian region researchers classified 7% of their subjects as DD patients^37^. In KSA it has also recently been estimated that around one-third of young diabetic patients suffer from atypical forms of diabetes^38^. Common symptoms of DD include obesity, insulin resistance, type of latent autoimmune diabetes in youth (LADY)^39^, autoantibodies, namely GAD56, IA2 and insulin antibodies, in T1D^40^. DD can be a major event during the young onset (11–19 years old) of diabetic patient, as a result of weight gain and insulin resistance caused as side effects of insulin treatment^41^. A potentially positive family history and increased BMI (>85^th^ percentile) could be considered as clinical measures to recognise such DD cases from those with visible T1D^37^. It is necessary that DD phenotype is appropriately managed in terms of both the diagnosis and therapeutic approach as it is extremely hard for these patients, who are usually mainly identified during paediatric ages^42^. DD appears to be an independent and potential risk factor for patients with T1D in gaining macro- and micro-vascular diseases^36^. Microvascular diseases in DD demonstrate an elevated risk for nephropathy and retinopathy, while macrovascular comorbidities include the metabolic syndrome^36^. There is still a lack of awareness for metabolic comorbidities and essential efforts are needed in order to recognise these patients and find strategies to decrease the rate of metabolic Syndrome in T1D^36^. An accurate treatment including a mixture of lifestyle indicators and sufficient insulin is required for these patients in order to boost their glycemic control and inhibit diabetes-associated complications^37^. Lifestyle behavioural modification changes, such as dietary and physical activity plans, may be suitable towards the prevention/management of both T1D and T2D^39^.

## Understanding double diabetes

### Worldwide prevalence

Since 1991, there was evidence of a ‘third’ type of diabetes—i.e. DD—which was originated by the observation that insulin deficiency and insulin resistance co-exist, but its classification was problematic by the lack of accurate measurement of the latter^43^. However, the first epidemiological data together with heredity observations made clinicians believe that around 4% of all T1D patients have the potential to also have T2D^43^. With the increase of obesity worldwide that has been observed over the last two decades^44^, among children with T1D ~25% suffered from excess body weight, which could be correlated with poor diabetic control, instable levels of blood glucose and elevated insulin dosage^45^. More recently, Merger et al. (2016) also confirmed the same percentage (25.5%) among T1D patients, which exhibited even higher macrovascular-associated comorbidities, such as coronary heart disease and stroke, increased microvascular conditions, both independently of glucose control. In another study including 200 patients with youth onset diabetes, 7% (mean age of 22.2 years and mean BMI of 29.8) were categorised in DD, with 29% still under the unknown category^37^.

#### The example of Middle East

In KSA, the rapid economic growth in the last 40 years and the heightened adoption of the westernised lifestyle has led to unhealthy dietary patterns and reduced physical activity^46^. Global estimates have shown that KSA belongs to the top ten countries with the highest current and projected diabetes prevalence worldwide (second highest in the Middle-East^47^. Specifically, it is the fourth country worldwide in terms of the incident rate of T1D (33.5 per 100 000 people)^48^. The diabetes burden in the Saudi society is still on the rise, suggesting that the more people being diagnosed with diabetes, the more will be at risk of developing hypertension, heart disease, stroke, kidney disease, blindness, amputations, dental disease and nervous system disease^49^. Additionally, it has been shown that almost one third of the young (12–20 years old) diabetic patients in KSA demonstrate both T1D and T2D clinical characteristics, therefore revealing atypical forms of DD^38^. A recent study was published determining the clinical and biological characteristics of DD among the Saudi youth, at a small scale including one clinic (n=312) and investigated a number of demographic, social, family and risk factors^38^. Furthermore, while diabetes risk factors in children and adolescents have been investigated for T1D and T2D in KSA^21^, additional studies are still needed for DD in the young population (11–19 years old).

### Pathophysiology

Diabetes, including DD, is not a simple, single clinical entity, but comprises a rather broad, mixed range of complex pathophysiological disease features.

#### Impaired immunity

Immunoglobulins can indicate the status of humoral immunity and are produced by B cells as a response to inflammatory diseases, such as diabetes^50^. Early on it was shown that T1D patients have a significantly lower serum concentration of immunoglobulin G (IgG), but comparable serum concentration of immunoglobulin A and M (IgA and IgM) with control non-diabetic subjects^51^. Nevertheless, a few T1D patients with onset in adolescence (<15 years old) were found completely deficient for IgA and IgG, indicating a more complicated mechanism^51^. On the other hand, population-based studies in T2D adult patients also show reduced IgG and IgM, but elevated IgA and immunoglobulin E (IgE) concentration, proposing immunoglobulins as valuable predictive indicators for T2D^50,52^. This immunoglobulin deficiency make T2D patients more prone to specific infections^53^, and quantification of this impaired immune response in DD will shed light to the exact mechanisms involved, as such studies in DD do not yet exist.

#### Autoimmunity

In T1D pathogenesis cellular autoimmune pathways cause destruction of insulin-secreting β-cells in the pancreas, resulting in inflammation^54^. Autoantibody positivity has been proposed as a predictive marker for estimating diabetes progression^55^. However, as mentioned above, distinct classification of diabetic patients is not always possible. In as early as 1993, it was recognised that there was a subset of non-insulin dependent patients with a later adult-onset of diabetes that also slowly developed latent autoimmune insulitis over the years, as revealed by analysing GAD autoantibodies^56^. In another study characterising autoimmunity in a young T2D population, it was found that, while the frequency of autoimmunity was significantly lower in T2D compared to T1D, there were still 8.1%, 30.3% and 34.8% of T2D children and adolescents testing positive for ICAs, GADs and IAAs, respectively, even without ever being managed with an insulin treatment^57^. Similarly, β-cell autoantibodies were detected in a subcategory of T2D children and adolescents, demonstrating LADY^58^. In addition, the detection of such autoantibodies, against not only β-cells but also self-reactive T-cells, in older patients brought about the clinical condition of latent autoimmune diabetes of the adult^59^. This is not be completely unexpected as the two main environmental determinants in T2D, namely diet and physical activity, can directly influence the expression of immune genes and the levels of systemic immune/inflammatory factors, such as interleukin 1β (IL-1β) and tumour necrosis factor α (TNFα) involved in the development of obesity^60^. It has been suggested that islet autoantibody assessment should be part of the T2D diagnostic evaluation, not only for predicting disease progression but also for distinguishing this pathogenically different T2D phenotype^61^. Additionally, black and white children with insulin-treated diabetes showed T2D-related symptoms in terms of obesity, irrespectively of their autoimmunity, further highlighting the ethnicity-dependent, heterogeneous pathogenesis mechanisms^26^. Almost 25% of young (<21 years old) T1D patients show a minimum of one organ-specific autoantibody, with females being at elevated risk of multiple autoimmunity and with some autoantibodies like against transgluminase are associated with younger age^62^.

#### Insulin resistance

Obesity-associated insulin resistance is considered as a chronic inflammatory condition originated in fat tissue^63^; in fact, based on key criteria used to define autoimmune diseases, obesity-associated insulin resistance, and T2D itself, have been recently proposed to this category^64^. However, as early as in 1986, researchers studied the natural progression of insulin resistance also in T1D patients, and demonstrated that most T1D patients with long disease duration showed various degrees of insulin resistance^65^. Especially in T1D patients with microalbuminuria, the insulin sensitivity was found particularly high, and one of the causes for increased health risks of these patients, such as for the development of renal and cardiovascular diseases^66^. A similar outcome was observed in young T1D patients with a unique insulin resistance phenotype, impaired cardiopulmonary fitness and exercise capacity, potentially implying a different pathophysiology from T2D^67^. The importance of insulin sensitivity start to be highly recognised as a missing link in the treatment and prevention of T1D, together with regulation of autoimmunity^68^. To appropriately assess insulin resistance in T1D patients, an insulin-resistance-syndrome (IRS) score was created based on varying clinical factors, such as waist-to-hip ratio, hypertension, high-density lipoprotein cholesterol/triglyceride plasma levels and presence of T2D in the family^69^. However, insulin dose and the metabolic syndrome were not good predictors in a study with DD patients^70^. All this evidence led once again to the complex concept of DD and the involvement of elevated insulin resistance in T1D, implication of liver fat and lipid profile, and subsequent increase of cardiovascular disease risk^71^.

#### Cardiovascular disease

Cardiovascular disease is the most common death cause in diabetic patients, nevertheless little is known about T1D effects on cardiovascular risks in younger populations^67^. Following up the link between insulin resistance and cardiovascular disease risk, a study in 1998 concluded that family history of T2D mediates this risk in T1D patients, further highlighting the complex association involved^72^. More specifically, both female and male T1D patients, especially the ones with T2D family history, were found to have increased intima-media thickness of the carotid artery, a common factor of atherosclerosis; future studies will show the causality and predictive values of this marker^73^. In the case of DD patients, meaning obese T1D patients, long-term hyperglycaemia together with abnormal partitioning of lipids can possibly lead to the boost of atherothrombotic pathophysiological characteristics^74^.

### Risk factors

The development of a complex disease like DD can be caused by a variety of risk factors, including genetic, pathophysiological, environmental and lifestyle.

#### Genetic predisposition

It is well known that T1D has sound genetic elements, with the main genetic susceptibility region belonging to the human leukocyte antigen (HLA) class II genes in chromosome 6, and more than 40 other non-HLA genetic markers being confirmed^75^. Furthermore, family history of T1D can significantly increase the risk of developing autoantibodies in childhood^76^. However, early investigations pointed towards the involvement of certain HLA haplotypes also in non-insulin dependent diabetes, proposing HLA as the main genetic cause of glucose intolerance in both T1D and T2D^77^. The influence of genetics is also evident in the reported higher chances of developing diabetes amongst first-degree consanguinity, where the genetic sharing is higher^78^. Apart from T1D, T2D is considered a complex genetic disease, with strong environmental triggers, consisting of various metabolic conditions, all linked with glucose intolerance and damage to insulin secretion^79^. Following genome-wide association studies that are considered the most promising in discovering novel disease markers^80^, the genetic architecture of T2D has been well-characterised^81^. A polymorphism in the potassium voltage-gated channel subfamily J member 11 (KCNJ11) gene in a case-control study in KSA^82^, and polymorphisms in the adenosine binding cassette transporter 1 (ABCA1) gene in a meta-analysis^83^, were significantly associated with T2D as they were observed more frequently in T2D patients. Additionally, polymorphisms in the fat mass and obesity-associated (FTO) gene, the so-called ‘obesity gene’, has been recognised to be involved in the progression of insulin resistance and presence of T2D in obese patients^84^, as well as responsiveness to dietary, exercise and drug-based weight loss interventions^85^. Finally, to the best of our knowledge no genetic study exists thus far regarding DD, however it would be very interesting to investigate the specific combination of polymorphisms that DD patients carry.

#### Obesity

The role of obesity in T2D is well-known and considered the single best predictor, with the vast majority of T2D patients (>85%) being overweight or obese leading to higher cardiovascular risks due to this uncontrolled weight gain^86^. These patients have increased pressure on the ability of their body to use insulin effectively to control blood sugar levels, therefore they are at increased risk of developing diabetes^87^. While in the Epidemiology of Diabetes Complications Study the prevalence of being overweight in T1D was found lower compared to the general population^88^, more recently in a sample of >2,700 T1D patients, depending on the diabetes duration, 20–25% were overweight and 6–10% were obese, with BMI status not being significantly associated with insulin dose and intensity of insulin treatment^89^. In children it was soon evident that both girls and boys developing T1D were taller and heavier during childhood than healthy children—mainly more overweight, but not more obese^90^ with every 10% weight increase resulting in 50–60% T1D risk increase before the age of three years^91^. Similarly, in another study with almost 12,000 children and adolescents (3–19 years old), the prevalence of overweight (but again not obese) in young T1D patients was higher compared to the non-diseased group^92^. These results were also confirmed in population-based studies in Norway and other European countries, where there was a weak, but significant, almost linear association between weight at birth and elevated risk of developing T1D in early childhood^93^, with the role of infant feeding still unknown^94^. Meta-analysis of four studies also showed that observed childhood obesity, assessed at different ages ranging from 1 to 12 years old, led to double chances of subsequent T1D development^95^. This effect is likely to be mediated via elevated beta-cell stress caused by hyperinsulinemia and lower insulin sensitivity linked with quick linear growth and obesity^96^. Weight gain early in childhood was also suggested to predict the risk of islet autoimmunity in children having a T1D first-degree relative^97^. Overall, obesity is an associated and precipitating factor for the development of both T1D and T2D in children, meaning that it plays a critical role also in the progression of DD^42^.

### Diet

The diet of an individual influences the quantity of insulin produced in order to meet the body’s blood glucose targets and to maintain optimal levels of blood glucose. Dietary patterns have been well linked with diabetes pathology and metabolic syndrome, more specifically with T2D development and treatment, with such effects varying according to sex and ethnicity^98^. Together with other risk factors like sedentary lifestyle, increased intake of high-carbohydrate, low-macronutrient and energy-dense fast foods is considered amongst the most prominent T2D risk factors^99^. For example, very recently in the Whitehall II Study, scientists identified a specific dietary pattern, including high consumption of diet soft drinks, sugar-sweetened beverages, burgers, crisps, white bread and other snacks that was associated with insulin resistance and increased T2D risk after adjusting for a range of cofounders^100^. On the other hand, fibre-rich foods have unequivocally been associated with reduced obesity and T2D risk according to several observational studies^101^. Research has also suggested that a low-carbohydrate ketogenic diet^102^, increased whole-grain intake^103^, consumption of certain whole fruits like grapes, apples and blueberries^104^ and high consumption of green leafy vegetables in fact resulted in lowered T2D risk^105^ are effective in reducing the risk and disease effects of T2D. Lastly, while the role of diet is evident in T2D, in T1D the effects are smaller, however there has been evidence that short duration of breast feeding, early introduction of cow’s milk formula, a late start of gluten consumption as well as high milk consumption at one year of age are considered dietary risk factors for the initiation of beta-cell antibodies^106^. Overall, given the important role of increased BMI in the development of DD, dietary patterns is also a critical factor in DD, however more targeted investigations are still needed.

### Physical activity

Active lifestyle has proven to be associated with several benefits to human health and wellbeing. Key among these benefits is its positive effects on muscle growth, muscle glucose utilisation, insulin liver sensitivity and overall glycemic control^107^. In other words, an active lifestyle can contribute to improvement in glycemic control and insulin action, even in cases with a family history of diabetes^46^. Looking more closely into specific physical activity patterns of diabetic patients, it seems that the latter have significantly lower total activity counts compared to healthy individuals with normal glucose levels, and also seemed to be more sedentary during the afternoon hours^108^. A meta-analysis suggests that individuals living a sedentary lifestyle have a significantly higher T2D and metabolic syndrome risk, while physical activity contributes positively to preventing or delaying T2D progression either by affecting BMI or improving insulin sensitivity^109^. Research suggests that adequate physical activity can lead to delaying the development of long-term complications of diabetes like retinopathy, neuropathy, nephropathy as well as reduce the rate of progression of existing complications^109^. The joint position statement of the American Diabetes Association and the American College of Sports Medicine^110^ as well as the exercise guidelines of the American Heart Association^111^ recommend that T2D patients require to exercise no less than once every 48 h (three times a week) in order to manage insulin resistance and blood glucose levels. Furthermore, the positive effects of exercise on insulin resistance is likely to be lost 48–72 h of exercise^112^, and short, vigorous exercise bouts have shown to enhance insulin resistance in T2D patients^113^.

## Potential management & treatment

Thus far, the best approach for treating DD has not been agreed, however since insulin resistance and weight gain are the main clinical, pathophysiological features of this ‘mixed’ disease, successful treatment regimens should include optimal measures to tackle these^35^. For example, developing insulin titration approaches is necessary to ensure patients receive adequate doses of insulin to maintain glycemic control^114^. Also, introducing strategies for lifestyle changes is required to avoid weight gain, obesity and to maintain low insulin resistance. Enhancing high-quality research and education on diabetes mellitus in relation to dietary practice and physical exercise has the potential to significantly reduce its prevalence and adverse health effects in target populations^46^.

### Insulin dosage and titration

The current advanced strategies in tackling diabetes, such as motoring devices and insulin analogs have significantly upgraded the quality of life of T1D patients. However, especially in the young populations, poor glycemic control is still evident causing not only to short-term, but also chronic disease complications^115^. Physiological factors contributing to this ineffective glycemic control are partially associated with the expected hormonal changes occurring during puberty. For instance, there is a significant increase in insulin response to intravenous or oral glucose^116^ and a significant decrease in glucose disposal^117^ in children and adolescents. Therefore, to ensure adequate glycemic control, insulin dosages are often elevated, and need to be constantly adjusted to avoid poor control during later stages of puberty. Additionally, overweight DD patients with insulin resistance do not often have insulin doses that have been titrated to reach target levels resulting in suboptimal treatment. The safety and efficiency of a daily insulin titration regime in regulating HbA1c levels was recently demonstrated in T2D patients^114^.

### Metformin

Metformin (Glucophage) is an oral antihyperglycemic drug, which is used as the first line treatment for the prevention and management of T2D, both in adults and children, and particularly for obese patients showing hyperinsulinemia^118^. The drug helps T2D patients respond better to their own insulin; decreases the quantities of glucose absorbed by intestines; and reduces the amount of glucose produced by the liver (Gong et al., 2012). In most clinical settings around the world, when metformin in combination with diet and exercise fails to keep blood sugar on normal levels, other therapeutic interventions are adopted, such as bariatric surgery^119^. Interestingly, metformin was successfully tested for its improved efficacy in treating T1D adult and adolescent patients, for a joint treatment together with insulin injections^120^. Furthermore, adding metformin to insulin therapy significantly improved insulin resistance in patients with DD-similar profiles^121^.

### Behavioural modification and lifestyle intervention

It was soon evident that lifestyle changes are the key to the prevention and treatment of environmentally-induced T2D, mainly caused by the sedentary lifestyle and obesity, and were more effective than metformin alone^122^. Successful interventions to tackle increased BMI, increased (saturated) fat consumption and reduced physical activity have been reported, resulting in significant weight loss and 58% diabetes risk reduction^123^. Lifestyle interventions in individuals with elevated risk of T2D can introduce sustaining lifestyle changes that result in long-term prevention of T2D^124^. For example, in the Diabetes in Europe—Prevention using Lifestyle, Physical Activity and Nutritional Intervention (DE-PLAN) project it was shown that T2D prevention via lifestyle intervention at a real-life primary health care setting provided by well-trained nurses led to beneficial long-term (3-year follow-up) outcomes, including modest reduction of weight, cardiovascular risk factors and overall diabetes risk^125^.

### Conclusions and future directions

As shown above, ‘hybrid’ DD is a complex phenomenon, demonstrating characteristics of both T1D and T2D, which is often misdiagnosed or ignored. There has been a rise in its prevalence, which is associated with the co-current increase of T2D due to the adoption of a ‘westernised’ lifestyle with sedentary behaviour and increased consumption of fat-based diets. Appropriate assessment of diabetes is necessary for early and correct diagnosis, which can be only achieved by increasing awareness of DD among general populations and primary care physicians. Additional research in DD patients in high-risk populations, such as in Middle East, is necessary to further document the health risks and symptoms of these patients, in order to develop appropriate, successful, long-term therapy regimens to treat them.
